# CPR knowledge in future physicians: evidence to strengthen undergraduate medical training

**DOI:** 10.3389/fmed.2025.1630153

**Published:** 2025-09-12

**Authors:** Gustavo Peña-Pinedo, Iván Hernández-Patiño

**Affiliations:** Instituto de Investigaciones en Ciencias Biomédicas, Universidad Ricardo Palma, Lima, Peru

**Keywords:** basic cardiac life support, medical students, undergraduate medical education, associated factors, knowledge

## Abstract

**Introduction:**

Out-of-hospital cardiac arrest (OHCA) is a leading cause of sudden death worldwide. In such cases, Cardiopulmonary Resuscitation (CPR), understood not only as chest compressions and ventilations but also as early recognition of cardiac arrest, activation of emergency services, and timely use of an Automated External Defibrillator (AED), is crucial for improving survival. These skills are especially important for medical students nearing the end of their academic training.

**Objective:**

To assess adult CPR knowledge levels and associated factors among pre-medical students at a private university.

**Methods:**

Observational, analytical, and cross-sectional study. A validated virtual questionnaire based on the 2020 AHA guidelines was administered to 151 students in their 12th semester. General knowledge and domain-specific knowledge (cardiac arrest recognition, high-quality CPR, and AED use) were evaluated, along with their association with socioeeducational variables.

**Results:**

Only 41.06% demonstrated adequate CPR knowledge (mean score = 11.12/22). AED use was the most deficient domain (70.2% inadequate knowledge), followed by high-quality CPR (62.91%) and cardiac arrest recognition (60.93%). Female sex (aPR = 1.12; 95% CI: 1.01–1.26; *p* = 0.045) and prior completion of a Critical Patient course (aPR = 1.21; 95% CI: 1.07–1.35; *p* = 0.002) were significantly associated with higher knowledge. No significant associations were found with age, previous CPR training, or having healthcare worker relatives.

**Conclusion:**

Fewer than half of the pre-intern students possessed adequate adult CPR knowledge despite prior training. Integrating periodic, competency-based training (addressing cognitive, practical, and attitudinal dimensions) into medical curricula could strengthen these skills and ensure future physicians are prepared to save lives.

## 1 Introduction

Cardiac arrest is, without a doubt, one of the most critical emergencies a healthcare professional may face. It is characterized by its sudden and unexpected onset, leaving a very narrow window of time for effective intervention. During this brief period, survival chances largely depend on whether someone nearby possesses the necessary knowledge to provide an adequate response (1). In this context, Cardiopulmonary Resuscitation (CPR) plays a fundamental role as part of the initial response to cardiac arrest (2) and represents the first line of defense in preserving the victim’s life until trained emergency medical personnel arrive. Therefore, mastering and teaching CPR is a cornerstone of medical education.

Out-of-hospital cardiac arrest (OHCA) continues to be a leading cause of death worldwide. In countries such as the United States, it is estimated that approximately 365,000 cases occur annually, with a survival rate of only about 10%, even after receiving care from emergency medical services (3).

Although recent national data on the incidence of OHCA en Peru are lacking, hospital-based reports in Lima recorded 4.0 and 2.56 cases per 1,000 emergency visits in 2004 and 2008, respectively (4). These figures suggest that the problema is present in our setting, although its actual scope has yet to be clearly established.

Nevertheless, it has been shown that bystander-performed CPR, when administered at the scene, can significantly improve survival rates, in some cases increasing them up to fourfold (5, 6). This evidence underscores the importance of training not only healthcare professionals but also those still in training—such as medical students—who can become key responders in emergency situations.

The American Heart Association (AHA) has established a “Chain of Survival” in which high-quality CPR plays a pivotal role, alongside early recognition of cardiac arrest, immediate activation of emergency medical services, and prompt use of an automated external defibrillator (AED) (2). Each step represents an opportunity to save lives, and knowledge of how to perform CPR should be considered a minimum standard among those preparing to become physicians.

However, various studies have shown that CPR knowledge among near-graduation medical students is often weaker than expected. In a multicenter study across 12 European countries, Baldi et al. (7) found that only 49.3% of final-year medical students could correctly identify cardiac arrest, despite previous training. In Peru, a national multicenter study reported that only 13% of evaluated students demonstrated adequate CPR knowledge (8).

Moreover, even individuals who have previously received CPR training often fail to retain what they have learned or feel unprepared to act, particularly when faced with assisting a stranger. This results in a gap between intention and real-world action (9). Several factors influence this level of knowledge, including prior experience with CPR, the frequency of training, the stage of medical education, and fears of legal consequences, such as being sued or causing unintentional harm (10). Cultural beliefs may also play a role; for example, reluctance to touch individuals of the opposite sex has been identified as a barrier to performing CPR in certain populations (11, 12). In addition, gender disparities persist: women are less likely to receive bystander CPR, especially in public spaces, a disparity linked to gender stereotypes, discomfort with physical contact, and limited female representation in training scenarios (13–16). This inequality translates into lower survival rates among women, even when CPR is administered (13, 14). Taken together, these findings highlight the need for comprehensive training that not only imparts technical knowledge but also promotes cultural sensitivity, gender equity, and proactive attitudes in emergency situations.

In light of this global context, it is relevant to analyze how CPR education is approached at the local university level, particularly in institutions responsible for training future healthcare professionals. At the School of Medicine of a private university in Lima, CPR content is included in the course “Critical Patient Management,” which is offered during the tenth semester. Until 2023, this was an elective course, meaning that not all students received CPR training during their undergraduate education.

Literature has showns that introducing CPR training early in medical school significantly improves both theoretical understanding and practical skill development, while also increasing students´ confidence and willingness to acti in emergency situations (17–19). However, early exposure alone is insufficient if not reinforced throughout the academic journey. This reinforces the importance of assessing students in the final stage of their training, as they are on the verge of becoming licensed physicians and assuming full responsibility for patient care. At this stage, CPR knowledge should not be viewed merely as part of the curriculum, but as a core clinical competency—one that can mean the difference between life and death.

Accordingly, the objective of this study was to determine the level of adult CPR knowledge among pre-intern medical students at a private university. Additionally, it aimed to analyze whether sociodemographic or academic factors are significantly associated with higher or lower levels of competency in this area.

## 2 Materials and methods

### 2.1 Study design

An observational, analytical, and cross-sectional study was conducted.

### 2.2 Population and sample

The study population consisted of 320 medical students enrolled in the twelfth semester of a private university in Lima, Peru. These students were approaching the start of their clinical internship in 2024. Data collection was carried out in December 2023.

The minimum sample size was calculated using a 95% confidence level, a 5% margin of error, and an expected prevalence of 20%, based on the study by Mejía et al. (8). This yielded a minimum required sample of 140 participants. Ultimately, 151 students were included using a non-probabilistic convenience sampling method.

### 2.3 Variables and instruments

The primary variable—level of adult CPR knowledge—along with its specific components, was categorized as either “adequate” or “inadequate” based on the total score obtained on a questionnaire. This classification was established through statistical analysis of the distribution of results within the sample using STATA v16.0, which enabled the definition of a data-driven cutoff point rather than relying on an external arbitrary standard.

The independent variables analyzed included age, sex, prior CPR training, having family members working in healthcare, and having completed the “Critical Patient Management” course.

Data were collected using a self-administered virtual questionnaire based on the 2020 American Heart Association (AHA) guidelines. The instrument was adapted from the questionnaire developed by Chuquihuanca and Liza (20), validated by expert judgment, and demonstrated high reliability (Cronbach’s alpha = 0.845). It consisted of 22 multiple-choice questions (one correct answer and three distractors), distributed across three dimensions:

- Recognition of cardiac arrest and activation of emergency medical services (items 1–7).
- High-quality CPR (items 8–16).
- Use of automated external defibrillator (AED) (items 17–22).

Each correct response was worth one point, for a total possible score ranging from 0 to 22.

### 2.4 Statistical analysis

Data were collected via Google Forms and exported to Microsoft Excel 2021 for initial organization. Subsequent statistical analysis was performed using STATA v16.0. Descriptive statistics were used to report frequencies and percentages for categorical variables, and measures of central tendency and dispersion for continuous variables.

Inferential analyses included the Chi-square test for categorical variables and the Mann–Whitney U test for comparing non-parametric distributions. Finally, a Poisson regression model with robust variance was used to calculate both crude and adjusted prevalence ratios (PR) with 95% confidence intervals. A *p*-value of <0.05 was considered statistically significant.

### 2.5 Ethical considerations

The study was approved by the Research Ethics Committee of the School of Medicine at the aforementioned private university (Code PG 1732023-A). Prior to completing the survey, participants provided informed digital consent, included at the beginning of the questionnaire. Anonymity and data confidentiality were guaranteed in accordance with the principles of the Declaration of Helsinki and the ethical principles of autonomy, beneficence, non-maleficence, and justice.

## 3 Results

A total of 151 medical students completed the survey, with a median age of 24 years (interquartile range: 23–25). The sample was predominantly female, with 106 participants (70.2%) identifying as women and 45 (29.8%) as men. More than half of the students, 83 (54.97%), reported having attended a CPR course, and among them, 50 (60.24%) had received this training within the past 2 years.

When asked whether they had a family member working in the healthcare field, 84 students (55.63%) responded affirmatively. Of these, the majority—70 students (83.33%)—had received some form of CPR training. However, only 17 (24.29%) attributed their CPR knowledge directly to their relatives. Additionally, approximately two-thirds of the participants, 101 (66.89%), had completed the Critical Patient course (Table 1).

**Table 1:** Socioeducational characteristics of medical students (*n* = 151).

Characteristics	*n*	%
Age	24 (23–25)*	
Sex		
Male	45	29.8
Female	106	70.2
Attended any CPR course?		
No	68	45.03
Yes	83	54.97
Time since attending the course		
> 4 years ago	18	21.69
3–4 years ago	15	18.07
≤ 2 years ago	50	60.24
Family member in the healthcare field		
No	67	44.37
Yes	84	55.63
Did the healthcare-relative receive CPR training?		
No	14	16.67
Yes	70	83.33
Did the healthcare-relative teach you CPR?		
No	53	75.71
Yes	17	24.29
Completed the “Critical Patient Management” course?		
No	50	33.11
Yes	101	66.89

*median (interquartile range).

Source: Authors’ elaboration.

Regarding adult CPR knowledge (Table 2), approximately 4 out of 10 students—62 participants (41.06%)—achieved an adequate level of knowledge, with a mean score of 11.12 out of 22 points. The highest score obtained was 21, and the lowest was 5.

**Table 2:** Level of knowledge in adult CPR and its dimensions among medical students.

Level of knowledge in adult CPR	*n*	(%)
CPR knowledge score (numerical)	11.12 (± 2.93)*	
CPR knowledge score		
Inadequate (5 to 11 points)	89	(58.94)
Adequate (12 to 21 points)	62	(41.06)
Cardiac arrest recognition and EMS activation (numerical)	4.19 (± 1.25)*	
Cardiac arrest recognition and EMS activation		
Inadequate (1 to 4 points)	89	(60.93)
Adequate (5 to 7 points)	59	(39.07)
High-quality CPR (numerical)	4.15 (± 1.68)*	
High-quality CPR		
Inadequate (0 to 4 points)	95	(62.91)
Adequate (5 to 9 points)	56	(37.09)
Use of AED (numerical)	2.76 (± 1.41)*	
Use of AED		
Inadequate (0 to 3 points)	106	(70.2)
Adequate (4 to 6 points)	45	(29.8)

*mean (standard deviation).

CPR, Cardiopulmonary Resuscitation; EMS, Emergency Medical Services; AED, Automated External Defibrillator.

Source: Authors’ elaboration.

When analyzing the specific components of CPR, 59 students (39.07%) demonstrated adequate knowledge in recognizing cardiac arrest and activating the emergency response system. Additionally, 56 (37.09%) knew how to perform high-quality CPR, and 45 (29.8%) were knowledgeable about the use of an automated external defibrillator (AED).

Significant differences in CPR knowledge were observed based on sex and participation in the Critical Patient Management course (Table 3). Female students showed higher levels of CPR knowledge compared to males (46.23% vs. 28.89%; *p* = 0.048). Similarly, those who had taken the Critical Patient Management course outperformed their peers who had not (49.5% vs. 24%; *p* = 0.003).

**Table 3:** Bivariate analysis of socioeducational characteristics and CPR knowledge level among medical students.

Socioeducational characteristics	Level of knowledge		*p*-value
	Inadequate (*n* = 89)	Adequate (*n* = 62)	
	*n* (%)	*n* (%)	
Age	24 (23–25)	24 (23–25)	0.3186^†^
Sex			0.048*
Male	32 (71.11)	13 (28.89)	
Female	57 (53.77)	49 (46.23)	
Attended CPR course			0.332*
No	43 (63.24)	25 (36.76)	
Yes	46 (55.42)	37 (44.58)	
Family member in healthcare			0.615*
No	41 (61.19)	26 (38.81)	
Yes	48 (57.14)	36 (42.86)	
Completed critical patient management course			0.003*
No	38 (76)	12 (24)	
Yes	51 (50.5)	50 (49.5)	

^†^Mann–Whitney U test used, significance level *p* < 0.05.

*Chi-square test of independence used, significance level *p* < 0.05.

Source: Authors’ elaboration.

Bold values indicate statistical significance at *p* < 0.05.

Two key factors associated with adequate CPR knowledge were identified (Table 4). In the unadjusted model, female students had a 13% higher prevalence of adequate knowledge compared to males (PR = 1.13; 95% CI: 1.01–1.28; *p* = 0.042), a result that remained significant after adjusting for confounding variables (aPR = 1.12; 95% CI: 1.01–1.26; *p* = 0.045). Additionally, having completed the Critical Patient Management course was consistently associated with a 21% higher prevalence of adequate knowledge, both in the crude (PR = 1.21; 95% CI: 1.07–1.35; *p* = 0.002) and adjusted model (aPR = 1.21; 95% CI: 1.07–1.35; p = 0.002).

**Table 4:** Crude and adjusted Poisson regression model: factors associated with adequate adult CPR knowledge among medical students.

Characteristics	Crude analysis			Adjusted analysis*		
	PR	95% CI	*p*-value**	PR	95% CI	*p*-value**
Sex						
Male	Ref.			Ref.		
Female	1.13	1.01–1.28	**0.042**	1.12	1.01–1.26	**0.045**
Attended CPR course						
No	Ref.			Ref.		
Yes	1.05	0.94–1.18	0.332	1.01	0.90–1.13	0.882
Family member in healthcare						
No	Ref.			Ref.		
Yes	1.02	0.92–1.15	0.890	1.05	0.95–1.18	0.286
Completed critical patient management course						
No	Ref.			Ref.		
Yes	1.21	1.07–1.35	**0.002**	1.21	1.07–1.35	**0.002**

*Adjusted for all covariables in the model.

**Statistically significant *p*-value < 0.05.

PR, Prevalence Ratio. 95% CI, 95% confidence interval.

Source: Authors’ elaboration.

Bold values indicate statistical significance at *p* < 0.05.

Conversely, other variables did not demonstrate statistically significant associations. Prior CPR training showed no relevant effect (aPR = 1.01; 95% CI: 0.90–1.13; *p* = 0.882), nor did having family members in the health sector (aPR = 1.05; 95% CI: 0.95–1.18; *p* = 0.286).

## 4 Discussion

A significant challenge was identified in medical education: less than half (41.06%) of final-year medical students demonstrated an adequate level of theoretical knowledge regarding adult CPR. This finding is concerning, as many of these future physicians may hesitate or feel unprepared when confronted with a cardiac arrest, where each minute without effective CPR decreases the chance of survival by 7 to 10% (2).

While our study provides relevant evidence on this issue, the findings should be interpreted in light of certain strengths and limitations. Among the strengths is the use of a validated instrument based on the 2020 AHA guidelines, which assessed three key dimensions of CPR knowledge. However, due to the cross-sectional design and convenience sampling, causal relationships cannot be established, nor can the results be generalized to other populations.

Additionally, the gender imbalance among final-year medical students—where women were overrepresented—may have skewed the results in favor of the female group, potentially influencing the observed association between being female and having higher CPR knowledge. The use of a self-administered online questionnaire also raises the possibility that some participants may have searched for answers while completing it. Furthermore, this study focused exclusively on theoretical knowledge, without assessing practical skills or attitudinal factors, which also play a crucial role in responding to cardiac arrest. Finally, the limited availability of automated external defibrillators (AEDs) in the university environment may have negatively impacted students’ scores related to AED use.

### 4.1 Overall level of CPR knowledge

Although the majority of medical students reported having received some form of CPR training—either through extracurricular courses (54.97%) or via the “Critical Patient Management” curricular course (66.89%)—58.94% exhibited inadequate knowledge of adult CPR. This finding reflects a trend similar to that observed in other parts of the world. For instance, Willmore et al. (21) found that although 80% of medical students across 21 British universities had received some CPR-related training during their studies, only 10.3% were able to correctly answer all the questions. This can be explained by the fact that many curricula restrict CPR instruction to isolated or short-term courses without periodic reinforcement, which negatively impacts knowledge retention. There is strong evidence indicating that, over months or years without regular practice or ongoing evaluations, students tend to forget what they have learned (1, 22). Moreover, the absence of mandatory retraining programs contributes to a gradual erosion of knowledge, preventing it from becoming consolidated (23). Therefore, it is crucial to improve teaching methodologies, increase the frequency of hands-on practice, and implement regular assessments to ensure that future physicians not only learn but retain and effectively apply adult CPR when most needed.

In addition to these curricular factors, the impact of the COVID-19 pandemic on medical students’ learning—especially regarding CPR knowledge—must be considered. During this period, universities were forced to transition abruptly to virtual education, which limited access to in-person training and simulation-based classes—essential for reinforcing theoretical knowledge. This situation may have negatively affected the level of knowledge observed in the students evaluated, as CPR is a competency that requires integration of knowledge, observation, and action. A recent study reported that 82% of medical students did not feel competent to apply their knowledge after attending virtual classes, and 76% believed that online learning failed to adequately replace face-to-face instruction (24).

While theoretical knowledge can be maintained, at least partially, in digital environments (25), virtual learning may impair knowledge retention if not supplemented with in-person practice or direct interaction. In this regard, Gupta et al. (26) compared two groups of medical students who received CPR training: one via virtual instruction and the other through in-person sessions. Although both groups acquired theoretical concepts, only those who participated in hands-on training performed better in both knowledge assessments and the quality of chest compressions. This supports the idea that the lack of in-person reinforcement during the pandemic may have hindered the consolidation of critical CPR knowledge. While online learning remains a viable alternative, in-person training with real-time feedback offers added value in the acquisition of practical skills.

### 4.2 Dimensions of CPR knowledge

According to the AHA experts (2), adult CPR training requires not only practical skills but also a solid theoretical foundation to support sound decision-making under pressure. However, our analysis revealed notable knowledge gaps—particularly regarding AED use—where fewer than one-third of students (29.8%) demonstrated adequate knowledge, a pattern consistent with findings from India and Peru (27, 28). This is a cause for concern, given that early defibrillation, alongside high-quality CPR, is critical for improving survival following cardiac arrest (2). A plausible explanation may lie in the limited availability of AEDs in academic settings, restricted practice opportunities with real or simulated devices, and insufficient integration of AED instruction into the medical curriculum.

### 4.3 Factors associated with adult CPR knowledge

In our study, two factors were found to be significantly associated with an adequate level of knowledge regarding adult CPR: being female and having completed the Critical Patient course.

Although other variables, such as having a family member in the healthcare field, were not statistically significant, previous research suggests that the family environment may influence attitudes toward health education and even shape professional self-confidence. Jasmon et al. (29) reported that family expectations have a direct effect on self-efficacy and vocational exploration among medical students. Additionally, families have been cited as a frequent and trusted source of medical information among young adults (30). From this perspective, having a relative in the healthcare sector may encourage early exposure or informal stimulation toward topics such as CPR. Although this variable did not show a meaningful impact in our sample, it remains worth investigating—particularly if the goal is to gain a more comprehensive understanding of the motivational and contextual factors that support interest in resuscitation skills.

Regarding gender, female students demonstrated higher levels of knowledge than their male counterparts (aPR = 1.12; 95% CI: 1.01–1.26; *p* = 0.045). This finding is consistent with recent research. For example, Mudassar et al. (31) reported that female university students in Pakistan had greater knowledge and more favorable perceptions of CPR compared to males, which may increase their willingness to engage in training (*p* < 0.05). Similarly, in Egypt, Baklola et al. (32) found that female students scored higher on CPR knowledge, attributing this to a more positive attitude toward learning emergency skills and greater exposure to formal training. However, a study conducted in Jordan found no statistically significant gender differences, suggesting that other factors—such as curricular structure and teaching methodology—may play a more decisive role in CPR knowledge acquisition (33).

A potential explanation for the higher knowledge observed among female students could relate to a stronger commitment to health-related learning, higher empathy, and more active participation in educational activities. Additionally, women may perceive CPR as a vital skill for helping others, further motivating them to master and apply it effectively.

On the other hand, more than half of the future physicians (50.5%) who opted to take the “Critical Patient Management” course did not reach the expected level of CPR knowledge. However, those who did (49.5%) performed significantly better than peers who did not take the course (aPR = 1.21; 95% CI: 1.07–1.35; *p* = 0.002). Similar findings were reported by Castillo (34), Torres (35) y Álvarez (36), who also observed that the elective course “Critical Patient Management” taught at our medical school positively influenced CPR knowledge.

The effectiveness of this course may stem from its methodology, which typically includes clinical simulations, complex case resolution, and emergency training that requires rapid response capabilities. These strategies not only enhance theoretical knowledge but also promote the development of practical skills essential for effective emergency response (37). However, even when such educational approaches yield improvements in knowledge, their impact may diminish over time without mechanisms for sustained reinforcement. Previous studies have shown that, without periodic practice or retraining programs, CPR skills tend to deteriorate gradually (4, 38, 39).

Given this situation, it is advisable that medical curricula not only introduce CPR instruction early in training but also incorporate regular update sessions to ensure long-term retention of acquired skills. The AHA recommends renewing CPR certification every 2 years (4, 40), and several studies have demonstrated that competencies may decline within 6 to 12 months in the absence of practice or reinforcement (39, 41, 42). Therefore, we recommend implementing practical evaluations every semester, as well as annual or biennial recertification courses (43) using clinical simulations and active learning methodologies to ensure continuous training and preparedness for real-life emergencies.

## 5 Conclusion

Less than half of the students assessed demonstrated adequate knowledge of adult CPR, with the use of the AED being the area where most difficulties were observed. Female students and those who had taken the Critical Patient course performed better, suggesting that certain curricular components may positively influence CPR knowledge levels.

Unlike previous studies that focus solely on the prevalence of CPR knowledge, our study also explores academic and sociodemographic factors associated with knowledge levels, providing useful evidence to inform the redesign of CPR training at the undergraduate level. These findings support the need for more frequent, hands-on, and strategically timed training sessions throughout key stages of medical education, in order to better prepare future physicians to respond effectively to real-life emergencies.

### 5.1 Recommendations

1. Strengthen CPR training during undergraduate education. We recommend incorporating mandatory, recurring high-fidelity simulation modules—including AED use—into the medical curriculum.
2. Establish a periodic recertification system. All medical students should receive formal CPR updates every 12 to 24 months, and these activities should be recognized with academic credit as part of their training.
3. Maintain the Critical Patient Management course as a core curricular component. Given its positive impact on CPR knowledge, this course should remain a required subject in the medical school curriculum.
4. Organize institutional activities focused on emergencies. We suggest that the School of Medicine promote large-scale simulations and awareness campaigns on cardiopulmonary emergencies, involving students actively in the planning and execution of such initiatives to foster a culture of preparedness within the university community.
5. Implement a peer-teaching program. It is advisable to enable upper-year students with valid CPR certification to train their junior peers—or even high school students—as a strategy for skill reinforcement and social responsibility.
6. Introduce CPR training from the earliest semesters. CPR content should be taught progressively and sequentially throughout the undergraduate program, starting from the first years.

## Data Availability

The original contributions presented in the study are included in the article/supplementary material, further inquiries can be directed to the corresponding author/s.
